# Prevalence and Factors Associated with Kidney Stones in the Elderly Iranian Population: Findings from the Ardakan Cohort Study on Aging (ACSA)

**DOI:** 10.34172/aim.33337

**Published:** 2025-02-01

**Authors:** Ahmad Delbari, Mehrdad Farrokhi, Ahmad Mehri, Mehran Saberian, Mohadeseh Sadri, Mohammad Saatchi

**Affiliations:** ^1^Iranian Research Center on Aging, University of Social Welfare and Rehabilitation Sciences, Tehran, Iran; ^2^Health in Emergency and Disaster Research Center, University of Social Welfare and Rehabilitation Sciences, Tehran, Iran; ^3^Department of Epidemiology, School of Public Health and Safety, Shahid Beheshti University of Medical Sciences, Tehran, Iran; ^4^Department of Biostatistics and Epidemiology, University of Social Welfare and Rehabilitation Science, Tehran, Iran

**Keywords:** Aging, Elderly population, Kidney stones

## Abstract

**Background:**

The incidence of kidney stones has been rising globally, particularly among the elderly. This study aims to determine the prevalence of kidney stones and its associated factors in Iran.

**Methods:**

This cross-sectional study was conducted using the data collected in the first phase of Ardakan Cohort Study on individuals aged 50 years and above, in the Yazd province, Iran. Baseline data was collected using a comprehensive checklist and kidney stone occurrence was ascertained through participants’ self-reported response to the question "Have you ever been diagnosed with kidney stones?" Logistic regression analysis was used due to the binary nature of the outcome.

**Results:**

Period prevalence of kidney stone was 22.79 (95% CI=19.41 to 24.12). Thus, out of the total sample of 4,884 individuals, 1113 participants had a history of kidney stones. Among these, 412 (37%) were female and 701 (63%) were male. Significant associations were observed between a history of kidney stones and factors such as self-reported poor health, alcohol consumption, dysuria, higher education level, male gender, and presence of calcium oxalate crystals.

**Conclusion:**

This study shows the high prevalence of kidney stones compared to other studies. Considering the relationship between kidney stones and some preventable disorders in the elderly, such as high blood pressure and alcohol consumption, it seems that paying attention to kidney stone disorders can be part of preventive health and treatment interventions for this population group.

## Introduction

 Kidney stones are a prevalent health issue affecting a considerable number of individuals worldwide and the elderly population, in particular, has experienced an increasing prevalence of kidney stones in recent years.^1^ In urinary tract problems, kidney stone disorder is one of the most common stones after urinary tract infections and prostate disorders.^2^ The prevalence of kidney stones varies across different regions. In Asia, reported prevalence ranges from 1% to 5%, in Europe, it is approximately 5% to 9%, and in North America, it ranges from 7% to 15%.^3^ Saudi Arabia has a prevalence close to 20%, while in China, it affects 4% of the general population.^4^ In the United States, the prevalence of kidney stones has tripled from 3.2% in 1980 to 9.6% in 2020.^5^ The prevalence of this disorder in the elderly in Asian countries has also varied from 4 to 10 percent.^6^ The latest studies conducted in the elderly population in some countries have also shown that the prevalence of this disorder in Chinese elderly is 6.4%^7^ and in 9.2 in South Korea.^7,8^

 Iran has also witnessed an increasing prevalence of kidney stones, particularly among the elderly, presenting a significant health concern. The reported prevalence of this disorder in Iran is 4.2 per thousand people.^9^ In a study in Iran, the prevalence of kidney stones in people over 40 years old was reported to be about 20%, and the elderly were part of this age group.^10^ With the rapid growth of the elderly population in Iran, it is estimated that by 2050, one-third of the population will be over 60 years old.^11^ This demographic shift has profound implications for healthcare in Iran, as age-related conditions like kidney stones become more prevalent.^12^ Consequently, it is crucial to gain insights into the prevalence and associated factors of kidney stones among the elderly population in Iran to develop effective prevention and treatment strategies.^13^

 Despite the substantial impact of kidney stones on quality of life,^14^ the condition remains underdiagnosed and undertreated in many countries, including Iran. This may be attributed to a lack of awareness among healthcare providers and patients regarding the importance of early detection and management.^15^ Hence, raising awareness about kidney stone disease among healthcare providers and patients is crucial for improving diagnosis rates and delivering timely treatment to the elderly population affected by this condition in Iran.

 However, kidney stone disease is increasingly prevalent among elderly Iranians due to various factors, including dehydration, low fluid intake, obesity, diabetes mellitus, hypertension, metabolic disorders, and dietary habits.^13^ Understanding these risk factors can facilitate the development of effective prevention strategies, ultimately enhancing the quality of life for elderly Iranians affected by this condition. While studies have examined the prevalence and risk factors of kidney stones in Iran, data from large cohort studies can provide a more accurate understanding of the prevalence of this condition specifically among the elderly population. Thus, this study aims to investigate the prevalence of kidney stones and explore associated factors in the Ardakan Cohort Study on Aging (ACSA).

## Materials and Methods

###  Study Design and Population

 The current cross-sectional study was conducted on people over 50 years old and older using ACSA data, in Ardakan, Yazd province, Iran, in 2019.^16^ The study adhered to the ethical principles outlined in the Helsinki Declaration for medical research involving human subjects.^17^ Data collection for this study took place from 2019 to 2021 during the initial phase of the cohort study. A sample size of 4884 participants was determined to ensure sufficient statistical power to detect associations between kidney stone occurrence and key predictors, with a confidence level of 95% and an estimated effect size based on previous cohort data. This sample size enables robust statistical comparisons and allows for subgroup analyses within demographic and clinical variables of interest.

###  Tools and Measurements

 Demographic characteristics of participants was collected using a questionnaire designed for the Ardakan Cohort Study. Kidney stone occurrence was defined as the presence of solid deposits comprised of minerals and salts in the kidneys, causing Considerable pain and discomfort during passage through the urinary tract.^18^ Participants were asked to self-report whether they had received a medical diagnosis of kidney stones. Childhood socioeconomic status (SES) was assessed using the question, “What was your SES during childhood, categorized into five levels based on your place of residence?” Participants were grouped into low, moderate to low, moderate, moderate to high, and high groups.^19^ Self-rated health was evaluated by participants rating their overall health on a scale ranging from very good to very bad, providing insight into their subjective perception of their health status.^20^ The Hospital Anxiety and Depression Scale (HADS) questionnaire was used to measure anxiety, where scores below seven were classified as “normal,” eight to ten as “mild or borderline,” and above ten as indicating an “anxiety disorder”.^21^ Health-related quality of life (SF12) questionnaire was used to measure the quality of life. Smoking status was determined by asking participants whether they had ever smoked at least once in their lifetime. Alcohol consumption was assessed through the question, “Have you ever consumed alcohol?”. addiction was evaluated by asking participants if they had ever used addictive substances. Dysuria, characterized by pain or discomfort during urination, was determined using the question, “Do you currently experience dysuria?” The presence of calcium oxalate crystals in urine was determined through laboratory analysis of urine samples. The physical activity of the participants was measured using the Physical Activity Scale for the Elderly (PASE).

###  Statistical Analysis

 Quantitative variables were summarized using mean and standard deviation, and qualitative variables were presented as counts and percentages. To compare groups, we used independent t-tests (for continuous variables) and chi-square tests (for categorical variables), with ANOVA employed for group comparisons of quantitative outcomes. The normality and homogeneity of variance assumptions were assessed using the Shapiro-Wilk test and Levene’s test, respectively. Assumptions were verified to ensure no expected cell count was less than 1, and that at most 20% of expected cell counts were less than 5. Linearity in the logit was assessed for quantitative predictors by examining the Box-Tidwell test. Logistic regression was used to analyze associations with kidney stones as a binary outcome. Backward selection was employed with an alpha level of 0.05 for variable removal. To address potential discrepancies between the Akaike information criterion (AIC) and the Bayesian information criterion (BIC), the models were compared, and priority was given to the model with the lowest BIC for parsimony. Logistic regression was employed for the analytical portion of the study, considering the binary outcome. Using the backward method, the final model is determined. In this way, non-significant variables were removed from the model and reduced models were selected using BIC and AIC statistics. Statistical analyses were performed using the Stata software version 14 with a confidence level of 95%.

## Results

###  Baseline Characteristics

 As presented in Table 1, the prevalence of kidney stone was 22.79 (95% CI = 19.41 to 24.12). Thus, out of the total sample of 4,884 individuals, 1113 participants had a history of kidney stones. Among them, 412 (37.02%) were women, while 701 (62.98%) were men. The highest proportion of individuals with previous kidney stones was observed in the 60-69 age group (43.31%). In terms of hypertension, a higher percentage of individuals in the kidney stone history group (52.92%) had high blood pressure compared to the group with no such history (49.28%). Regarding self-rated health, the majority of participants in both groups (57.61%) rated their health as moderate to good. Approximately half of the participants in both groups (49.21%) had a middle SES. Dysuria was reported by 15.2% of participants, and 21.8% of individuals had a positive result for calcium oxalate crystals.

**Table 1:** Demographic Profile of Study Participants (N = 4884)

**Variable**	**Levels **	**Non-kidney Stone** **3771(77.21%)**		**Kidney Stone** **1113 (22.79%)**		**Total** **(N=4884)**		***P*** ** Value**
		**No.**	**%**	**No.**	**%**	**No.**	**%**	
Age group	50-59	1573	41.71	445	39.99	2018	41.32	0.358
	60-69	1424	40.41	482	43.31	2006	41.07	
	70-79	567	15.04	159	14.29	726	14.86	
	> = 80	107	2.84	27	2.43	134	2.74	
Gender	Female	2123	56.30	412	37.02	2535	51.90	< 0.001
	Male	1648	43.70	701	62.98	2349	48.10	
Marital status	Single	361	9.57	81	7.28	442	9.05	0.019
	Married	3410	90.43	1032	92.72	4442	9095	
Education level	Illiterate	549	14.59	137	12.31	686	14.07	< 0.001
	Elementary (reference)	1887	50.13	460	41.33	2347	48.12	
	Middle school	525	13.95	187	16.80	712	14.60	
	High school	429	11.40	150	1348	579	11.87	
	College	374	9.94	179	16.08	553	11.34	
Job status	Not working/ disabled	2374	63.07	790	70.98	3164	64.88	< 0.001
	Working	819	21.76	223	20.04	1042	21.37	
	Other/homemaker	571	15.17	100	8.98	671	13.76	
BMI	≤ 25	824	21.85	206	18.51	1030	21.09	0.016
	≥ 25	2947	78.15	907	81.49	3854	78.91	
High blood pressure	No	1912	50.72	524	47.08	2436	49.89	0.033
	Yes	1858	49.28	589	52.92	2447	50.11	
Self-rated health	Very good	147	3.91	41	3.69	188	3.86	0.142
	Good	1106	29.39	329	29.61	1435	29.44	
	Middle to somewhat good	2189	58.17	619	55.72	2808	57.61	
	Bad	263	6.99	102	9.18	365	7.49	
	Very bad	58	1.54	20	1.80	78	1.60	
Self-rated socioeconomic status	High level	13	0.35	7	0.63	20	0.41	0.367
	Middle to high level	270	7.21	68	6.15	338	6.97	
	Middle level	1851	49.45	535	48.37	2386	49.21	
	Middle to low level	910	24.31	294	26.58	1204	24.83	
	Low level	699	18.68	202	18.26	901	18.58	
Anxiety status	Normal	2780	74.33	813	73.46	3593	74.17	0.846
	Borderline-middle	520	13.90	161	14.58	681	14.06	
	Abnormal	440	11.76	130	11.78	570	11.77	
Sleep quality	Poor quality	928	24.61	269	24.17	1197	24.51	0.764
	Good quality	2843	75.39	844	75.83	3687	75.49	
Smoking status	Never	2928	77.91	748	67.57	3676	75.56	< 0.001
	Former	370	9.85	154	13.91	524	10.77	
	Current	460	12.24	205	18.52	665	13.67	
Alcohol use	No	3744	99.63	1093	98.74	4837	99.42	0.001
	Yes	14	0.37	14	1.26	28	0.58	
Addiction	No	3583	95.34	1033	93.32	4616	94.88	0.007
	Yes	175	4.66	74	6.68	249	5.12	
Dysuria	Never	3251	86.65	871	78.61	4122	84.81	< 0.001
	A little	397	10.58	176	15.88	573	11.79	
	Moderate	78	2.08	49	4.42	127	2.61	
	Much/very much	26	0.69	12	1.08	38	0.78	
Calcium oxalate crystals status	No	2994	79.40	825	74.12	3819	78.19	< 0.001
	Yes	777	20.60	288	25.88	1065	21.81	
**Variable**	**Count**	**Non-kidney Stone** **3771(77.21%)**		**Kidney Stone** **1113 (22.79%)**		**Total (N=4884)**		***P*** ** Value**
		**Mean (95% CI)**	**SD**	**Mean (95% CI)**	**SD**	**Mean (95% CI)**	**SD**	
Quality of life (mental)	Count	49.22 (48.90-49.54)	10.02	49.45 (48.87-50.03)	9.98	49.27 (48.99-49.56)	10.01	0.25
Quality of life (physical)	Count	45.70 (45.39-46.02)	9.91	45.57 (44.98-46.15)	10.00	45.68 (45.40-45.95)	9.93	0.66
Physical activity	Count	136.55 (133.86-139.26)	84.60	137.141 (131.94-142.33)	88.24	136.69 (134.29-139.08)	85.44	0.42
BMI	Count	28.53 (28.36-28.70)	5.00	28.62 (28.33-28.91)	4.65	28.55 (28.40-28.69)	4.92	0.31

###  Factors Associated with Kidney Stones

Table 2 displays the results of univariable and multivariable analyses, adjusted for other variables, examining the associations between each variable and the presence of kidney stones. The odds ratio of having kidney stones was significantly higher in males compared to females (Adjusted odds ratio [OR] = 2.06, 95% confidence interval [CI]:1.77 to 2.40). Individuals with high blood pressure had 1.27 times higher odds of having kidney stones compared to those without high blood pressure (95% CI:1.10 to 1.46). Poor self-reported health status was associated with increased odds ratio of kidney stones compared to moderate to good health status (Adjusted OR = 1.33, 95% CI:1.03 to 1.72). A significant positive relationship was found between alcohol consumption and a history of kidney stones (Adjusted OR = 2.65, 95% CI:1.24 to 5.65). Additionally, a significant association was observed between kidney stones and a positive status for calcium oxalate crystals (Adjusted OR = 1.38, 95% CI = 1.18 to 1.62). Participants reporting moderate to mild dysuria had 2.355 times higher odds of having kidney stones compared to those without dysuria (95% CI = 1.615 to 3.434).

**Table 2:** Relationship Between Each of the Demographic Characteristics with Having a History of Kidney Stones in Crude and Adjusted form Using Univariable and Multivariable Logistic Regression

**Variables**	**Levels**	**Crude OR**	**95% CI Crude OR**		***P*** ** value**	**Adjusted OR**	**95% CI Adjusted OR**		***P*** ** Value**
			**Lower**	**Upper**			**Lower**	**Upper**	
Age	Count	0.99	0.97	1.01	0.771	-	-	-	-
Gender	Female(reference)	1	-	-	-	1	-	-	-
	Male	2.19	1.91	2.51	< 0.001	2.06	1.77	2.40	< 0.001
Marital status	Single(reference)	1	-	-	-	-	-	-	-
	Married	1.35	1.04	1.73	0.019	-	-	-	-
Education level	Illiterate	1.02	0.82	1.26	0.830	1.007	0.808	1.255	0.945
	Elementary (reference)	1	-	-	-	1	-	-	-
	Middle school	1.46	1.20	1.77	< 0.001	1.301	1.060	1.597	0.012
	High school	1.43	1.16	1.77	0.001	1.197	0.956	1.499	0.115
	College	1.96	1.60	2.41	< 0.001	1.608	1.285	2.012	< 0.001
Job status	Not working/ disabled (reference)	1	-	-	-	-	-	-	-
	Working	0.81	0.69	0.96	0.020	-	-	-	-
	Other/homemaker	0.52	0.41	0.66	< 0.001	-	-	-	-
BMI	≤ 25 (reference)	1	-	-	-	-	-	-	-
	≥ 25	1.23	1.03	1.45	0.016	-	-	-	-
High blood pressure	No(reference)	1	-	-	-	1	-	-	-
	Yes	1.15	1.01	1.32	0.033	1.27	1.10	1.46	0.001
Self-rated health	Very good	0.98	0.61	1.41	0.940	0.77	0.534	1.126	0.182
	Good	1.051	0.90	1.22	0.65	0.92	0.785	1.086	0.340
	Middle to somewhat good (reference)	1	-	-	-	1	-	-	-
	Bad	1.37	1.07	1.75	0.012	1.33	1.03	1.72	0.027
	Very bad	1.21	0.72	2.04	0.75	1.16	0.68	1.98	0.55
Self-rated socioeconomic status	High level	2.13	0.82	5.56	0.119	-	-	-	-
	Middle to high level (reference)	1	-	-	-	-	-	-	-
	Middle level	1.14	0.86	1.52	0.340	-	-	-	-
	Middle to low level	1.28	0.95	1.72	0.100	-	-	-	-
	Low level	1.14	0.84	1.56	0.377	-	-	-	-
Anxiety status	Normal (reference)	1	-	-	-				
	Borderline-middle	1.05	0.87	1.28	0.563	-	-	-	-
	Abnormal	1.010	0.81	1.24	0.924	-	-	-	-
Sleep quality	Poor quality (reference)	1	-	-	-	-	-	-	-
	Good quality	1.02	0.87	1.19	0.764	-	-	-	-
Smoking status	Never(reference)	1	-	-	-				
	Former	1.62	1.32	1.99	< 0.001	-	-	-	-
	Current	1.74	1.45	2.09	< 0.001	-	-	-	-
Alcohol use	No (reference)	1	-	-	-	1	-	-	-
	Yes	3.42	1.62	7.20	0.001	2.65	1.24	5.65	0.012
Addiction	No (reference)	1	-	-	-	-	-	-	-
	Yes	1.46	1.10	1.94	0.007	-	-	-	-
Dysuria status	Never (reference)	1	-	-	-	1	-	-	-
	A little	1.65	1.36	2.00	< 0.001	1.60	1.31	1.96	< 0.001
	Moderate	2.34	1.62	3.37	< 0.001	2.35	1.61	3.43	< 0.001
	Much/very much	1.72	0.86	3.42	0.121	1.73	0.86	3.51	0.123
Calcium oxalate crystals status	No (reference)	1	-	-	-	1	-	-	-
	Yes	1.34	1.52	1.57	< 0.001	1.38	1.18	1.62	< 0.001
Quality of life (mental)	Count	0.99	0.99	1.00	0.680	-	-	-	-
Quality of life (physical)	Count	1.00	0.99	1.00	0.502	-	-	-	-

## Discussion

 The objective of this study was to determine the prevalence and associated factors of kidney stones among the elderly population in the center of Iran. Considering that we calculate the history of kidney stones during a person’s lifetime as prevalence, in fact, in this study, we are obtaining the lifetime prevalence of kidney stone. The results showed that almost a fifth of the participants had a history of kidney stones during their lifetime.

 Comparing these findings to estimates from other countries, the prevalence observed in this study appears higher than reported rates in the United States (5.2%)^22^ and China (7.2%),^23^ while varying prevalence rates have been reported in other studies conducted in these countries.^7^ Similar to our study, another study in Saudi Arabia reported a prevalence consistent with our findings.^24^ Studies by Moftakhar et al,^10^ Rafiei et al,^25^ and Moludi et al^26^ also found comparable prevalence. also, Dehghani et al^27^ reported a higher prevalence, while variations among studies can be attributed to differences in study populations and timeframes. Considering that kidney stones were self-reported in all studies and the focus was on the elderly population, the observed prevalence represents a lifetime prevalence, which may explain the higher rates among the elderly. Since high heat and sweating are related to having kidney stones,^28^ and the place where the study was conducted in Yazd province is a hot region, the high prevalence of kidney stones in this study may be the reason.

 The results indicated a significantly higher prevalence of kidney stones in men compared to women, which aligns with findings from studies by Scales et al,^12^ Bihl and Meyers,^29^ and Moftakhar et al.^10^ Several factors contribute to the higher prevalence in men. Anatomical differences, such as longer urethras, can impede the passage and expulsion of small stones. The presence of the prostate gland in men can also obstruct urine flow, increasing the risk of stone formation.^30^ Hormonal factors, specifically elevated testosterone levels in men, can lead to increased calcium excretion in urine, contributing to stone formation.^31^ Dietary choices, including animal protein and sodium, which is more common among men, can elevate the risk of stone formation. These dietary patterns result in higher levels of calcium and uric acid in urine, which are major components of kidney stones.^32^ Although kidney stones are more prevalent in men, they can still occur in women, and individual lifestyle choices and medical conditions can influence of developing kidney stones.^33^

 Our study identified a significant association between high blood pressure and kidney stones. This finding is consistent with a study by Shlipak et al, which demonstrated a potential link between high blood pressure, kidney dysfunction, and the increased formation of kidney stones in the elderly.^34^ Another study in the United States found a direct relationship between the prevalence of high blood pressure and elevated serum creatinine levels, which can elevate the risk of kidney stone development.^35^ While kidney stones and high blood pressure are not directly causative, certain factors contribute to the presence of both conditions. Dietary patterns characterized by high sodium or animal protein intake, which are common risk factors for kidney stones, can also increase the risk of high blood pressure.^36^ Additionally, dehydration, a known risk factor for kidney stone formation, can contribute to high blood pressure.^33,37^ It is important to acknowledge that while these factors may be associated with both conditions, they do not necessarily imply a causative relationship. In the elderly population, the risk of hydration disorders and kidney stone development may be heightened among individuals at risk of high blood pressure.^38^

 Although, results showed that there is a relationship between alcohol consumption and kidney stones, in contrast to this study, other studies showed that there is no relationship between alcohol consumption and kidney stone. A study conducted in China has shown that the alcohol consumption can be one of the protective factors for kidney stones.^39^ In a meta-analysis, no clear effect of alcohol has been demonstrated.^40^ The results of a cohort study in Japan show that alcohol consumption not only does not play a risk factor, but also plays a role in the prevention of kidney stones. Despite such studies, the relationship between alcohol consumption and kidney stones is that excessive or chronic alcohol consumption can potentially increase the risk of having kidney stones.^41^ Since the present study was conducted in an elderly population, the direct association between alcohol consumption and kidney stones could be due to the cumulative effect of alcohol consumption seen over a lifetime.

 In this study, having moderate and mild dysuria was related to having a history of kidney stones. Hoffman has shown in her study that dysuria has a significant relationship with kidney stones.^42^ Lam et al proposed a range of clinical symptoms of the disease may be seen in kidney disorders that kidney stones and dysuria can occur together.^43^ However, it is important to note that dysuria can have multiple causes, and kidney stones are just one potential cause.^44^ Other conditions such as urinary tract infections, prostate problems, or inflammation of the urethra can also cause dysuria.^45^ Dysuria in the elderly can be a symptom of kidney stones, but it is not the sole indicator.^46^ Proper evaluation and diagnosis by a healthcare professional are important to determine the exact cause and initiate appropriate treatment.

 In terms of limitations, the present study is a cross-sectional study and it is not clear whether related factors caused the occurrence of kidney stones or vice versa. Kidney stone measurement was self-reported and the results may be affected by under-reporting or over-reporting. However, this study shows a better clinical picture of kidney stones in the elderly in a large sample size. On the other hand, the relationship of each variable was analyzed in an adjusted manner to determine the relationship size of each variable more precisely.

## Conclusion

 In conclusion, this study provides valuable insights into the prevalence and associated factors of kidney stones among the elderly population. The findings indicate that approximately one-fourth of the elderly participants had a history of kidney stones, with high blood pressure, self-reported health status, alcohol consumption, and education level identified as significant factors associated with this condition. These factors can be targeted and managed through lifestyle modifications and social interventions. Given the multifaceted nature of health in old age, it is imperative to prioritize the management of kidney stones and the control of related risk factors. This study highlights the importance of not only focusing on medical and therapeutic interventions but also addressing lifestyle factors and implementing strategies to control the identified risk factors.

## References

[1] Geraghty RM, Proietti S, Traxer O, Archer M, Somani BK (2017). Worldwide impact of warmer seasons on the incidence of renal colic and kidney stone disease: evidence from a systematic review of literature. J Endourol.

[2] Delfan B, Baharvand-Ahmadi B, Bahmani M, Mohseni N, Saki K, Rafieian-Kopaei M (2015). An ethnobotanical study of medicinal plants used in treatment of kidney stones and kidney pain in Lorestan province, Iran. J Chem Pharm Sci.

[3] Aune D, Mahamat-Saleh Y, Norat T, Riboli E (2018). Body fatness, diabetes, physical activity and risk of kidney stones: a systematic review and meta-analysis of cohort studies. Eur J Epidemiol.

[4] Ma RH, Luo XB, Li Q, Zhong HQ (2018). Systemic analysis of urinary stones from the Northern, Eastern, Central, Southern and Southwest China by a multi-center study. BMC Urol.

[5] Mao W, Hu Q, Chen S, Chen Y, Luo M, Zhang Z (2021). Polyfluoroalkyl chemicals and the risk of kidney stones in US adults: a population-based study. Ecotoxicol Environ Saf.

[6] Liu Y, Chen Y, Liao B, Luo D, Wang K, Li H (2018). Epidemiology of urolithiasis in Asia. Asian J Urol.

[7] Zeng G, Mai Z, Xia S, Wang Z, Zhang K, Wang L (2017). Prevalence of kidney stones in China: an ultrasonography based cross-sectional study. BJU Int.

[8] Choi SY, Yoon CG (2017). Urologic diseases in Korean military population: a 6-year epidemiological review of medical records. J Korean Med Sci.

[9] Tadayyon F, Sabbagh M (2011). The prevalence of kidney stone different composition in patients referred to the lithotripsy wards. J Isfahan Med Sch.

[10] Moftakhar L, Jafari F, Ghoddusi Johari M, Rezaeianzadeh R, Hosseini SV, Rezaianzadeh A (2022). Prevalence and risk factors of kidney stone disease in population aged 40-70 years old in Kharameh cohort study: a cross-sectional population-based study in southern Iran. BMC Urol.

[11] Jahangiry L, Bagheri R, Darabi F, Sarbakhsh P, Naghibi Sistani MM, Ponnet K (2020). Oral health status and associated lifestyle behaviors in a sample of Iranian adults: an exploratory household survey. BMC Oral Health.

[12] Scales CD Jr, Smith AC, Hanley JM, Saigal CS (2012). Prevalence of kidney stones in the United States. Eur Urol.

[13] Luyckx VA, Tuttle KR, Garcia-Garcia G, Gharbi MB, Heerspink HJ, Johnson DW (2017). Reducing major risk factors for chronic kidney disease. Kidney Int Suppl (2011).

[14] Stern KL, Gao T, Antonelli JA, Viprakasit DP, Averch TD, Chi T (2019). Association of patient age and gender with kidney stone related quality of life. J Urol.

[15] Shlipak MG, Tummalapalli SL, Boulware LE, Grams ME, Ix JH, Jha V (2021). The case for early identification and intervention of chronic kidney disease: conclusions from a Kidney Disease: Improving Global Outcomes (KDIGO) Controversies Conference. Kidney Int.

[16] Aminisani N, Azimi-Nezhad M, Shamshirgaran SM, Mirhafez SR, Borji A, Poustchi H (2022). Cohort profile: the IRanian Longitudinal Study on Ageing (IRLSA): the first comprehensive study on ageing in Iran. Int J Epidemiol.

[17] World Medical Association (2013). World Medical Association Declaration of Helsinki: ethical principles for medical research involving human subjects. JAMA.

[18] Moe OW (2006). Kidney stones: pathophysiology and medical management. Lancet.

[19] Baigi V, Nedjat S, Fotouhi A, Janani L, Mohammad K (2016). Subjective social status in association with various health and socioeconomic indicators in Tehran. J Public Health.

[20] Nedjat S, Hosseinpoor AR, Forouzanfar MH, Golestan B, Majdzadeh R (2012). Decomposing socioeconomic inequality in self-rated health in Tehran. J Epidemiol Community Health.

[21] Montazeri A, Vahdaninia M, Ebrahimi M, Jarvandi S (2003). The Hospital Anxiety and Depression Scale (HADS): translation and validation study of the Iranian version. Health Qual Life Outcomes.

[22] Stamatelou KK, Francis ME, Jones CA, Nyberg LM, Curhan GC (2003). Time trends in reported prevalence of kidney stones in the United States: 1976-1994. Kidney Int.

[23] Wang W, Fan J, Huang G, Li J, Zhu X, Tian Y (2017). Prevalence of kidney stones in mainland China: a systematic review. Sci Rep.

[24] Ahmad F, Nada MO, Farid AB, Haleem MA, Razack SM (2015). Epidemiology of urolithiasis with emphasis on ultrasound detection: a retrospective analysis of 5371 cases in Saudi Arabia. Saudi J Kidney Dis Transpl.

[25] Rafiei H, Malekpoor F, Amiri M, Rahimi Madiseh M, Lalegani H (2014). Kidney stone development among older adults in Iran. J Indian Acad Geriatr.

[26] Moludi J, Fateh HL, Pasdar Y, Moradinazar M, Sheikhi L, Saber A (2022). Association of dietary inflammatory index with chronic kidney disease and kidney stones in Iranian adults: a cross-sectional study within the Ravansar non-communicable diseases cohort. Front Nutr.

[27] Dehghani A, Alishavandi S, Nourimajalan N, Fallahzadeh H, Rahmanian V (2022). Prevalence of chronic kidney diseases and its determinants among Iranian adults: results of the first phase of Shahedieh cohort study. BMC Nephrol.

[28] Vicedo-Cabrera AM, Goldfarb DS, Kopp RE, Song L, Tasian GE (2020). Sex differences in the temperature dependence of kidney stone presentations: a population-based aggregated case-crossover study. Urolithiasis.

[29] Bihl G, Meyers A (2001). Recurrent renal stone disease-advances in pathogenesis and clinical management. Lancet.

[30] Walsh C, Collyns T (2017). The pathophysiology of urinary tract infections. Surgery (Oxford).

[31] Gupta K, Gill GS, Mahajan R (2016). Possible role of elevated serum testosterone in pathogenesis of renal stone formation. Int J Appl Basic Med Res.

[32] Taylor EN, Stampfer MJ, Curhan GC (2004). Dietary factors and the risk of incident kidney stones in men: new insights after 14 years of follow-up. J Am Soc Nephrol.

[33] Sofia NH, Walter TM, Sanatorium T (2016). Prevalence and risk factors of kidney stone. Glob J Res Anal.

[34] Shlipak MG, Fried LF, Cushman M, Manolio TA, Peterson D, Stehman-Breen C (2005). Cardiovascular mortality risk in chronic kidney disease: comparison of traditional and novel risk factors. JAMA.

[35] Coresh J, Wei GL, McQuillan G, Brancati FL, Levey AS, Jones C (2001). Prevalence of high blood pressure and elevated serum creatinine level in the United States: findings from the third National Health and Nutrition Examination Survey (1988-1994). Arch Intern Med.

[36] Rodrigues FG, Lima TM, Zambrano L, Heilberg IP (2020). Dietary pattern analysis among stone formers: resemblance to a DASH-style diet. J Bras Nefrol.

[37] Khalili P, Jamali Z, Sadeghi T, Esmaeili-Nadimi A, Mohamadi M, Moghadam-Ahmadi A (2021). Risk factors of kidney stone disease: a cross-sectional study in the southeast of Iran. BMC Urol.

[38] Centers for Disease Control and Prevention (CDC). Chronic Kidney Disease in the United States, 2019. Atlanta, GA: US Department of Health and Human Services, Centers for Disease Control and Prevention. 2019. p. 3.

[39] Chewcharat A, Curhan G (2021). Trends in the prevalence of kidney stones in the United States from 2007 to 2016. Urolithiasis.

[40] Jones P, Karim Sulaiman S, Gamage KN, Tokas T, Jamnadass E, Somani BK (2021). Do lifestyle factors including smoking, alcohol, and exercise impact your risk of developing kidney stone disease? Outcomes of a systematic review. J Endourol.

[41] Ping H, Lu N, Wang M, Lu J, Liu Y, Qiao L (2019). New-onset metabolic risk factors and the incidence of kidney stones: a prospective cohort study. BJU Int.

[42] Hoffman A, Braun MM, Khayat M (2021). Kidney disease: kidney stones. FP Essent.

[43] Lam CW, Lan L, Che X, Tam S, Wong SS, Chen Y (2009). Diagnosis and spectrum of melamine-related renal disease: plausible mechanism of stone formation in humans. Clin Chim Acta.

[44] Herrera R, Orantes CM, Almaguer M, Alfonso P, Bayarre HD, Leiva IM (2014). Clinical characteristics of chronic kidney disease of nontraditional causes in Salvadoran farming communities. MEDICC Rev.

[45] Shaheen G, Akram M, Jabeen F, Ali Shah SM, Munir N, Daniyal M (2019). Therapeutic potential of medicinal plants for the management of urinary tract infection: a systematic review. Clin Exp Pharmacol Physiol.

[46] Mehta P, Leslie SW, Reddivari AK. Dysuria. In: StatPearls. Treasure Island, FL: StatPearls Publishing; 2019.

